# An Unusual Presentation of Subscapularis Tendon Calcific Tendonitis

**DOI:** 10.7759/cureus.48678

**Published:** 2023-11-12

**Authors:** Muath Alqahtani, Ahmed Jalal, Ahmad Alghamdi, Abdulrasheed Halawani, Mamdouh Masri

**Affiliations:** 1 Orthopedic Surgery, King Fahad General Hospital, Jeddah, SAU; 2 Orthopedic Surgery, King Abdulaziz Hospital, Makkah, SAU; 3 Orthopedic Surgery, East Jeddah Hospital, Jeddah, SAU; 4 Orthopedic Surgery, King Abdul-Aziz University Hospital, Jeddah, SAU; 5 Orthopedic Surgery, Dr. Suliman Fakeeh Hospital, Jeddah, SAU

**Keywords:** debridement, arthroscopy, tendinopathy, tendonitis, calcific, subscapularis

## Abstract

Rotator cuff tendons are predisposed to calcific tendonitis and may present in single or multiple tendons. Despite the existence of several treatment strategies that have been proposed, clinical outcomes are controversial. Our study aims to report a case of large subscapularis tendon calcific tendonitis that was refractory to non-operative management and to investigate the clinical presentation, imaging findings, and treatment outcomes. A 42-year-old male patient presented to us for the first time complaining of right shoulder pain and weakness for 18 months with no improvement with non-operative treatment. Shoulder radiographs showed calcific tendonitis of the supraspinatus tendon. Magnetic resonance imaging (MRI) showed a larger calcific deposit with low signal intensity on all sequences within the subscapularis, along with smaller calcific deposition within the supraspinatus tendon. The patient underwent right shoulder arthroscopic debridement of calcific tendonitis and has shown an improved range of motion and pain at postoperative follow-up. Considering arthroscopic debridement for calcific tendonitis that is refractory to non-operative treatment is an effective and safe intervention in relieving patient pain and improving function and quality of life.

## Introduction

Codman was the first to describe the presence of calcium deposits within the rotator cuff tendon [1]. Then, the "calcifying tendinitis" terminology was defined by Plenk in 1952 [2]. Calcific tendonitis is a painful shoulder condition characterized by single or multiple calcium hydroxyapatite crystal deposits within a pathologically healthy tendon in the rotator cuff. This differs from the calcification in degenerative tendinopathy, which is composed of a heterogeneous mixture of calcium salts diffusely scattered throughout the tendon in areas of collagen degeneration or tear [3].

Thus, the disease subsides spontaneously in most cases, and a group of patients continues to complain of pain and shoulder dysfunction, and calcific tendonitis does not show any signs of resolution [4]. The incidence ranges between 2.7% and 20%, and about 10-20% of patients have deposits present bilaterally. Most studies found the incidence in women is higher compared to men. In most studies, the average age was between 30 and 50 years, and calcific tendonitis was located mostly in the supraspinatus tendon and varies in its presentation in the infraspinatus tendon and is rarely in the subscapularis and teres minor [5]. This disorder’s etiology and treatment options remain controversial among orthopedic shoulder surgeons [6]. As a matter of fact, 35-50% of calcific tendonitis patients are symptomatic [6,7].

Conventional management used includes rest, ice, nonsteroidal anti-inflammatory (NSAID) drugs, physical therapy, and subacromial corticosteroid injections. However, strong evidence to support any of these treatment modalities is lacking [8]. Extracorporeal shock-wave therapy (ESWT) is an alternative noninvasive and less expensive modality for refractory pain in calcific tendonitis patients. Initially, it was used for lithotripsy to treat nephrolithiasis patients. In shoulder calcific tendinitis, shock waves are delivered transcutaneously with or without local anesthesia for 10 to 30 minutes but could require multiple sessions with a success rate reaching 65%. Bannuru et al.'s systematic review showed that high-energy ESWT is effective in shoulder function and pain in cases of chronic calcific tendonitis and can result in complete resolution of calcifications [9].

Operative management may be considered if a patient fails nonoperative modalities and has persistent chronic pain and functional impairment affecting their quality of life. While reviewing the literature, a high level of evidence could not be found specifying the duration of nonoperative modalities before stepping up to more invasive interventions [9,10].

Our study aims to report a case of large subscapularis tendon calcific tendonitis that was refractory to nonoperative management and to investigate the clinical presentation, imaging findings, and treatment outcomes.

## Case presentation

A 42-year-old male patient, employed as a school teacher, right-handed, and a never-smoker, presented to us for the first time with a complaint of right shoulder pain persisting for the past 18 months. Initially, the pain was sudden and dull in nature, worsened with overhead activity, and was not alleviated by over-the-counter analgesics or rest. The pain intensified in the two months prior to presentation and was accompanied by difficulty sleeping due to pain and tenderness over the anterior and lateral aspects of the affected shoulder. Upon clinical examination, no deformity, swelling, atrophy, discoloration, or scapular winging was observed. The primary finding was a painful range of motion without significant deficit, where forward flexion was 180 degrees, abduction was 160 degrees, external rotation was 60 degrees, and internal rotation reached up to the lumbar 3 (L3) vertebral level. Rotator cuff power tests, particularly the Jobe's, belly-press, and lift-off tests, were painful, obscuring an accurate power assessment of the rotator cuff. Impingement tests, including the Neer and Hawkins' tests, were positive, while biceps provocative tests, such as Speed's and Yergason's tests, were negative. The patient completed a questionnaire to determine the Disabilities of the Arm, Shoulder, and Hand (DASH) score, which was 66.4 for subsequent follow-up and assessment of the intervention.

**Figure 1:**
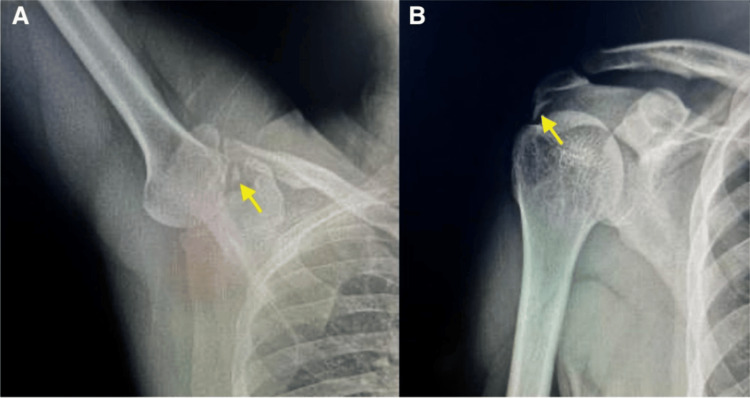
Right shoulder radiographs (A) axillary view showing calcific deposition within the anterior portion of the shoulder girdle (yellow arrow). (B) AP view showing the deposit in the subacromial space suggestive of calcific tendinitis within the supraspinous tendon (yellow arrow).

Right shoulder radiographs revealed calcific tendonitis in the subacromial space around the supraspinatus tendon insertion without evidence of adjacent subscapularis calcific tendonitis (Figure 1).

**Figure 2:**
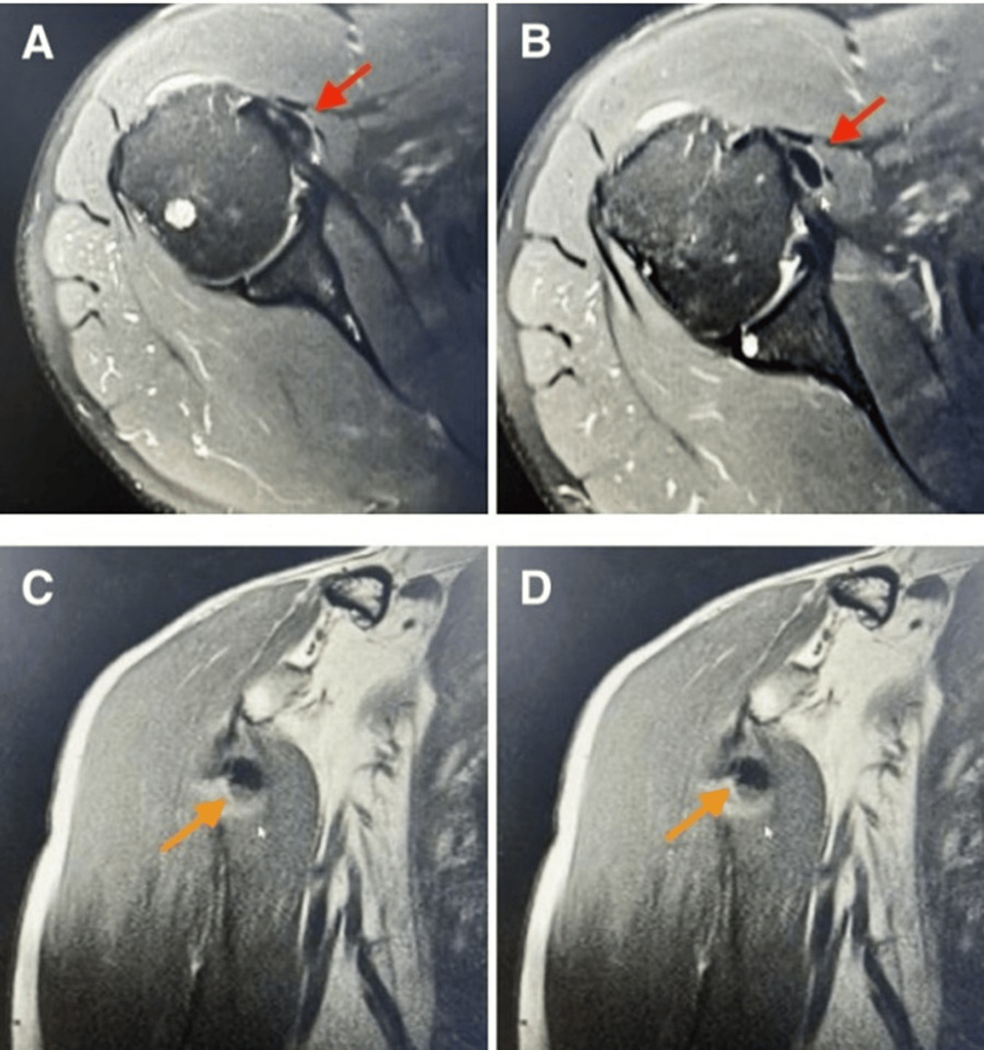
Right shoulder MRI (A and B) Axial cuts showing a large calcium deposit found within the substance of the subscapularis tendon measuring roughly 9 × 5 mm (red arrow). (C and D) Coronal cuts showing that the calcific deposit within subscapularis tends to be larger (orange arrow).

Upon the patient's persistent symptoms and chronic presentation, magnetic resonance imaging (MRI) was ordered, which revealed an isolated finding of large calcific tendonitis in the subscapularis tendon that was not apparent on the prior shoulder radiographs. This was in addition to the previously seen supraspinatus calcific tendonitis on radiographs (Figure 2).

No further pathology of the rotator cuff, bursa, labral, or ligaments was observed. Given the patient's clinical presentation and the chronicity of his condition, he was elected for right shoulder arthroscopic decompression and debridement of the calcium deposits from both the supraspinatus and subscapularis calcific tendonitis, which were refractory to nonoperative management, including a physical therapy program and NSAIDs.

**Figure 3:**
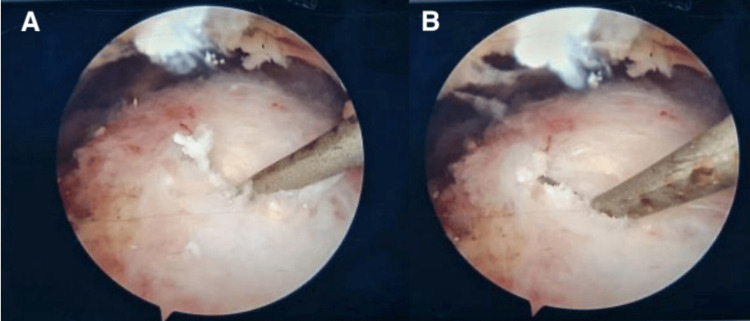
Right shoulder arthroscopic of the subacromial space (viewing form lateral) (A) The probe is shown piercing the layer covering the calcium deposit, and (B) the probe is facilitating the venting of calcium out of the anterior portion of the supraspinatus tendon.

**Figure 4:**
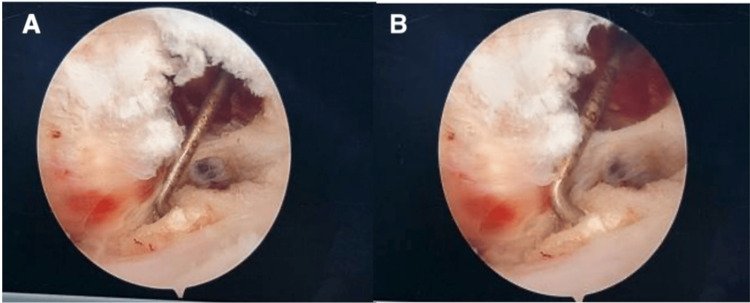
Right shoulder arthroscopic of glenohumeral joint (viewing from posterior postal while applying posterior level push) (A and B) Probing and venting of the calcium deposits located at the anterior surface of the upper one-third of the subscapularis tendon.

Diagnostic arthroscopy revealed calcific tendonitis calcium deposits within the substance of the superior one-third of the subscapularis tendon and the anterior part of the supraspinatus tendon near the rotator interval. Complete diagnostic arthroscopy revealed no further intra-articular pathology, except for an inflamed and thickened subacromial bursa and the identification of a Bigliani type 3 acromion morphology. Subsequent subacromial bursectomy was performed with the use of a shaver and electrocautery to debride the subacromial space and ensure adequate visualization of the index pathology, followed by acromioplasty using a 4 mm burr. The calcific tendonitis calcium deposits were decompressed using a probe to break a small window to vent the calcification out of the supraspinatus and subscapularis tendons without compromising their integrity (Figures 3, 4).

The procedure was completed with irrigation with an additional three bags of normal saline (5 liters/bag).

The patient followed the standard rotator cuff repair postoperative protocol, including immobilization for four weeks with active elbow ROM. At the four-week follow-up patient started supervised passive and active assisted ROM exercises and achieved a full ROM and endurance of his right shoulder at the six-month follow-up with improved pain, negative impingement tests, and prior symptoms had completely resolved along with an independent pain-free full active and passive range of motion and post-operative DASH score of 32.5.

## Discussion

Calcific tendonitis is a common disorder of the rotator cuff tendons with clinical manifestations that vary depending on the pathology stage In the precalcific stage, there are typically no symptoms. In the calcific stage, the patient may experience mild to moderate pain and stiffness. In the resorptive stage, the patient may experience severe pain, stiffness, and swelling. Surgical intervention is often required once nonoperative modalities have failed. While the etiology and pathogenesis of calcific tendinitis have a vast debate in the literature, some believe that impingement leads to degeneration of the rotator cuff tendons, followed by diseased tendon calcification; on the other hand, others believe that it is attributed to events of intra-tendinous hypoxia [11,12].

Arthroscopic treatment for calcific tendonitis proved to be effective management for chronic and refractory cases, which did not improve with conservative management [7]. Radiographs and MRI can be considered as initial investigation tools, but ultimately, the gold standard tool is arthroscopy [13].

Once conservative management does not yield satisfactory pain relief, arthroscopy should be considered, as it has been shown to be effective [13].

Subscapularis calcific tendonitis has been addressed in several reports. All cases involve patients with subscapularis calcific tendonitis who did not achieve optimal results with conservative management. In contrast, patients who underwent arthroscopic debridement reported pain relief at the final follow-up, along with favorable outcomes [7,14-19].

One report indicated that 91% (23) of patients with rotator cuff calcific tendonitis improved following an arthroscopic procedure and experienced positive outcomes [20].

## Conclusions

Arthroscopic debridement for refractory and chronic calcific tendonitis cases is an effective and safe intervention in relieving patient pain and improving function and quality of life. Initial management of nonoperative management is effective, but the literature lacks high-level evidence of unified nonoperative protocol. In cases of refractory cases, considering operative management for calcific tendonitis is an option to relieve patient pain, restore function, and improve quality of life.
